# A neural oscillatory signature of sustained anxiety

**DOI:** 10.3758/s13415-023-01132-1

**Published:** 2023-10-25

**Authors:** Ariel D. Roxburgh, David J. White, Christian Grillon, Brian R. Cornwell

**Affiliations:** 1 Monash Addiction Research Centre, Eastern Health Clinical School, Monash University, Melbourne, Australia; 2 Turning Point, Eastern Health, Melbourne, Australia; 3 Centre for Human Psychopharmacology, Swinburne University of Technology, Hawthorn, Australia; 4 National Institute of Mental Health, Bethesda, MD USA; 5 Centre for Mental Health, Swinburne University of Technology, Hawthorn, Australia

**Keywords:** Anxiety, Beta oscillation, Alpha oscillation, Magnetoencephalography, Sensorimotor, Intraparietal sulcus

## Abstract

**Background:**

Anxiety is a sustained response to uncertain threats; yet few studies have explored sustained neurobiological activities underlying anxious states, particularly spontaneous neural oscillations. To address this gap, we reanalysed magnetoencephalographic (MEG) data recorded during induced anxiety to identify differences in sustained oscillatory activity between high- and low-anxiety states.

**Methods:**

We combined data from three previous MEG studies in which healthy adults (total *N* = 51) were exposed to alternating periods of threat of unpredictable shock and safety while performing a range of cognitive tasks (passive oddball, mixed–saccade or stop-signal tasks). Spontaneous, band-limited, oscillatory activity was extracted from middle and late intervals of the threat and safe periods, and regional power distributions were reconstructed with adaptive beamforming. Conjunction analyses were used to identify regions showing overlapping spectral power differences between threat and safe periods across the three task paradigms.

**Results:**

MEG source analyses revealed a robust and widespread reduction in beta (14-30 Hz) power during threat periods in bilateral sensorimotor cortices extending into right prefrontal regions. Alpha (8-13 Hz) power reductions during threat were more circumscribed, with notable peaks in left intraparietal sulcus and thalamus.

**Conclusions:**

Threat-induced anxiety is underpinned by a sustained reduction in spontaneous beta- and alpha-band activity in sensorimotor and parietal cortical regions. This general oscillatory pattern likely reflects a state of heightened action readiness and vigilance to cope with uncertain threats. Our findings provide a critical reference for which to identify abnormalities in cortical oscillatory activities in clinically anxious patients as well as evaluating the efficacy of anxiolytic treatments.

**Supplementary Information:**

The online version contains supplementary material available at 10.3758/s13415-023-01132-1.

## Introduction

Anxiety symptoms commonly present across psychopathological diagnostic boundaries (American Psychiatric Association, 2013; Lanius et al., 2006; Nitschke & Heller, 2005), although those specifically diagnosed with anxiety disorders tend to experience more frequent and severe states of anxious arousal (American Psychiatric Association, 2013). In contrast to fear, which can be defined as a short-lived response to a perceived immediate threat, anxious arousal describes a more sustained response to threats that are uncertain and more psychologically and physically distal (Davis et al., 2010). Under these circumstances, the organism becomes cautious and hypervigilant toward the environment (Grillon, 2002; Grupe & Nitschke, 2013). While anxious arousal can serve an adaptive function when real threats materialize, it can lead to distress and impaired functioning when the state is persistent or extreme (American Psychiatric Association, 2013). Persistent hypervigilance is a hallmark of posttraumatic stress disorder (PTSD) and is associated with early exaggerated sensory-perceptual responding in patients with the disorder (Ge et al., 2011; Morgan & Grillon, 1999). Similarly, heightened sensory-perceptual responding can be seen in healthy participants during induced anxiety (Cornwell et al., 2007, 2017), indicating that experimentally induced anxiety can model aspects of clinical anxiety. However, human studies have predominantly focused on task- or stimulus-related activity and how anxiety—measured as a state or trait variable—modulates these activities (Cornwell et al., 2012). Few studies have investigated sustained, nontask-specific**,** state-related changes related to the induction of anxiety by a well-established anxiety-induction paradigm.

The threat-of-shock procedure, wherein participants are exposed to periods of threat (i.e., unpredictable shock) and safety, is a reliable way to induce anxiety that has been validated in preclinical, clinical, and pharmacological studies as an anxiety manipulation (Cornwell et al., 2017; Davis et al., 2010; Grillon, 2008; Robinson et al., 2013). Threat-of-shock has been used in conjunction with noninvasive neuroimaging to localize sustained changes during anxiety states (Alvarez et al., 2011; Balderston et al., 2017; Hasler et al., 2007; MacNamara & Barley, 2018; Vytal et al., 2014). For example, Andreatta et al. (2015) exposed participants to virtual reality settings associated with threat-of-shock or safe conditions and found threatening contexts were associated with changes in sustained fMRI-BOLD activity in emotional, navigational, and motor areas. Also, using fMRI, Vytal et al. (2014) found increased coupling between the amygdala and dorsal medial prefrontal cortex (dmPFC) during sustained threat periods, which has been reported in other studies (Bijsterbosch et al., 2015; Robinson et al., 2016). Using these regions as “seeds,” Vytal et al. (2014) further showed increased coupling between this seed network and areas involved in defensive responding (e.g., insula, OFC, dACC, basal ganglia, and thalamus) and decreased coupling with structures involved in emotional control (e.g. ITG). Using fMRI during periods of induced anxiety, several regions have shown increased activity, such as the insula, amygdala, basal ganglia, cingulate gyrus, orbital frontal cortex, bed nucleus of the stria terminalis (BNST), and right inferior frontal gyrus (Alvarez et al., 2011; Andreatta et al., 2015; McMenamin & Pessoa, 2015; Vytal et al., 2014). Although a preliminary picture of an extended brain network underlying sustained anxiety is emerging, the exclusive reliance on hemodynamic (i.e., fMRI) and metabolic (i.e., PET, SPECT) measurements presents problems. First, anxious arousal alters cerebrovascular function and often is accompanied by respiratory changes that alter arterial CO_2_ tensions and create changes in cerebral blood flow (Giardino et al., 2007). Thus, changes in cerebral activity measured by using fMRI between anxious and nonanxious conditions may be confounded by anxiety-induced changes in cardiovascular function. Second, fMRI scanning has been shown to be anxiogenic with its partly enclosed, acoustically stressful environment (Tessner et al., 2006), which could obscure any relative differences between threatening and safe contexts.

Electrophysiological studies of anxiety have focused predominantly on brain data in relation to task events or participant responses (Cornwell et al., 2012). Yet, such analysis might miss the sustained elements of anxiety that are task irrelevant. However, there has been some research into sustained anxiety using electrophysiological measurements, such as MEG and EEG. Frost et al. (1978) showed no changes in EEG alpha activity between participants in threatening and nonthreatening conditions. Most recent research reports asymmetry of EEG oscillatory power between hemispheres, which is thought to relate to emotion regulation (Goodman et al., 2013; Reznik & Allen, 2018; Verona et al., 2009). While informative, EEG provides low spatial resolution and may not be optimally sensitive to subtle region-specific changes to consolidate with fMRI findings. Few have probed the electrophysiological activity associated with sustained anxiety with MEG, which offers higher spatial resolution than EEG. A notable exception is Balderston et al. (2017) who used threat-of-shock to induce anxiety while collecting both MEG and fMRI data. The authors specifically targeted alpha (8-13 Hz) activity, finding threat-related reductions in the left intraparietal sulcus (IPS). Due to the IPS’s role in attention (Goltz et al., 2015; Molenberghs et al., 2007; Thakral & Slotnick, 2009), the authors suggested anxiety facilitates attentional processing.

It has been shown that conditioned fear is associated with increased theta-band (4-8 Hz), coupling between the midline frontal and amygdala regions, which is thought to reflect adjustments to uncertainty (Cavanagh & Shackman, 2015). Additional fear is associated with changes in theta-band power in the amygdala, hippocampus, and medial PFC (Lesting et al., 2011; Maratos et al., 2009; Pape et al., 2005). However, it is not clear whether theta-band changes would be shown during states of sustained anxious arousal. There also is evidence that beta oscillations (i.e., 14-30 Hz) might be modulated by induced anxiety. Beta has been extensively studied (Schmidt et al., 2019) and is thought to be important in action/thought stopping in the right IFG and basal ganglia (Castiglione et al., 2019; Swann et al., 2009; 2011; Wagner et al., 2017; Zavala et al., 2015) and readiness for action over sensorimotor areas (Kilavik et al., 2013). Furthermore, inhibitory deficits found in induced anxiety are associated with differences in beta oscillations (Cornwell et al., 2012; Roxburgh et al., 2020). Kilavik et al. (2013) suggest that sensorimotor beta increases are associated with motor stability, whereas decreases are associated with motor action and action readiness. Action readiness is likely what an adaptive anxious state would require, enabling an organism to quickly escape danger. Thus, anxiety might reduce sensorimotor beta activity to facilitate readiness for action.

We present a new analysis of spontaneous neural oscillatory activity from three different MEG studies employing threat of unpredictable shocks to induce sustained anxiety states, because this allows us to rule out effects that are nonspecific to anxiety, which might instead be related to a specific task (e.g., different tasks may recruit different levels of attention, decision making, and responding). With the same method of anxiety induction, we sought to identify common oscillatory correlates of anxious arousal across three different cognitive contexts: a passive listening auditory oddball task (oddball study; Cornwell et al., 2007), a mixed saccadic eye movement task (mixed-saccade study; Cornwell et al., 2012), and a stop-signal task (stop-signal study; Roxburgh et al., 2020). Such a variety of tasks combined with analyses that are not locked to task related events will provide a unique insight into the general neural-oscillatory attributes of sustained anxiety. Importantly, we specifically focused our analyses on intervals during threatening and nonthreatening periods in which an initial phasic fear response to the onset of threat has given way to a sustained state of anxious arousal. We hypothesize that these intervals during threatening periods are marked by decreased beta and alpha oscillatory activity in sensorimotor and parietal cortices, reflecting a state in which the individual is primed to rapidly respond to imminent danger. Whole-brain MEG analyses allowed us to examine whether other structures involved in affective processing (e.g., amygdala, hippocampus, medial prefrontal cortices) also show spontaneous oscillations that might underpin sustained anxiety. We also aim to explore theta oscillatory activity during sustained anxiety given theta activity has been implicated in fear.

## Methods and materials

### Participants

We selected three participant samples from previous studies that used similar threat of unpredictable shock procedures. The oddball (Cornwell et al., 2007) and mixed-saccade studies (Cornwell et al., 2012) were conducted at the National Institutes of Health (Bethesda, MD), and the stop-signal study (Roxburgh et al., 2020) was completed at Swinburne University of Technology (Hawthorn, VIC, Australia). In the oddball and mixed-saccade studies, we obtained MEG recordings and MR images from 20 and 17 healthy, adult volunteers, respectively. Because the present analysis was specifically designed to identify neural oscillatory correlates of anxious arousal, four participants from the oddball study were excluded for not reporting increased anxiety during threat of shock periods. In the stop-signal study, we obtained MEG recordings and MR images from 18 healthy, adult volunteers. All studies were approved by local ethics boards (Combined Neuroscience Institutional Review Board of the National Institutes of Health or Swinburne University Human Research Ethics Committee), and all participants provided written, informed consent before participation. Exclusion criteria were the same in all three studies (no past or current DSM-IV/V diagnosis, or current use of psychoactive or illicit drugs), except that the stop-signal study relied on self-report, whereas the other two used structured, clinical interviews for DSM-IV (First et al., 1995) and urine analysis to determine eligibility. The same participants removed for excessive head movement in the original studies also were excluded from the current study (14 participates total). The final sample comprised 51 participants (mean age 27 years, 35 males, 16 females). A breakdown of participant characteristics by study are shown in supplementary Table S1.

### Design and procedure

MEG data were recorded in two runs containing threatening (THREAT) and nonthreatening (SAFE) conditions.

#### Threat of shock procedure

THREAT and SAFE conditions alternated with counterbalancing of the starting condition. During THREAT, participants were informed that they could receive electric shocks at any time, whereas participants were told that they would not receive a shock during SAFE. Participants were not required to respond in any way to conditions, rather the conditions were present to moderate anxious states (induced anxiety or THREAT vs. SAFE). All three studies followed the same shock workup procedure before the beginning of the task to determine an appropriate shock level. The procedure involved an initial weak shock, which was followed by shocks of increasing amplitude after participant feedback, until a level rated as “moderately aversive/uncomfortable” was reached. Shocks (100 ms; 150 µs pulses every 10 ms) were delivered to the wrist of the nondominant hand by using a Digitimer DS7A constant current stimulator (Digitimer, LLC, USA; used in stop-signal) or Grass S88 constant voltage stimulator (Astro-Med, Inc., RI, USA; used in oddball and mixed-saccade) and were between 3 and 5 mA (oddball and mixed-saccade) or 0.5 mA and 5.3 mA (stop-signal). Participants received 8 shocks across the 2 runs combined, which were delivered on a fixed pseudorandom schedule (the oddball study used a single shock delivered at the end of the first run). The start of each context was signaled by a voice recording (oddball study) or a text message on the screen (mixed-saccade and stop-signal studies), indicating whether they were at risk of receiving shocks or safe for the following period. At the end of each run, participants rated their anxiety on a scale from 0 (“no anxiety”) to 10 (“extreme anxiety”) for each condition.

#### Task details

##### Passive oddball task

Three types of tones were presented binaurally: one standard tone (probability of 0.80) and two deviant tones (each with a probability of 0.10). Participants were not required to respond (passive listening task) (Cornwell et al., 2007).

##### Stop-signal task (SST)

A visual go stimulus (left or right facing arrow) was presented, to which participants responded with the correct corresponding button as quickly as possible. On a small portion of trials (probability 0.3), an auditory tone was presented after the visual stimulus indicating participants should withhold their responses (Roxburgh et al., 2020).

##### Mixed-saccade task

Participants were presented with a blue or red “+” sign, indicating whether to make a pro-saccade (eye movements toward a stimulus) or anti-saccade (eye movements away from a stimulus). A peripheral cue was then presented immediately following the offset of the instruction cue either to the left or right of a central fixation point, to which participants responded with the corresponding eye movements (Cornwell et al., 2012).

#### Procedure

**Fig. 1:**
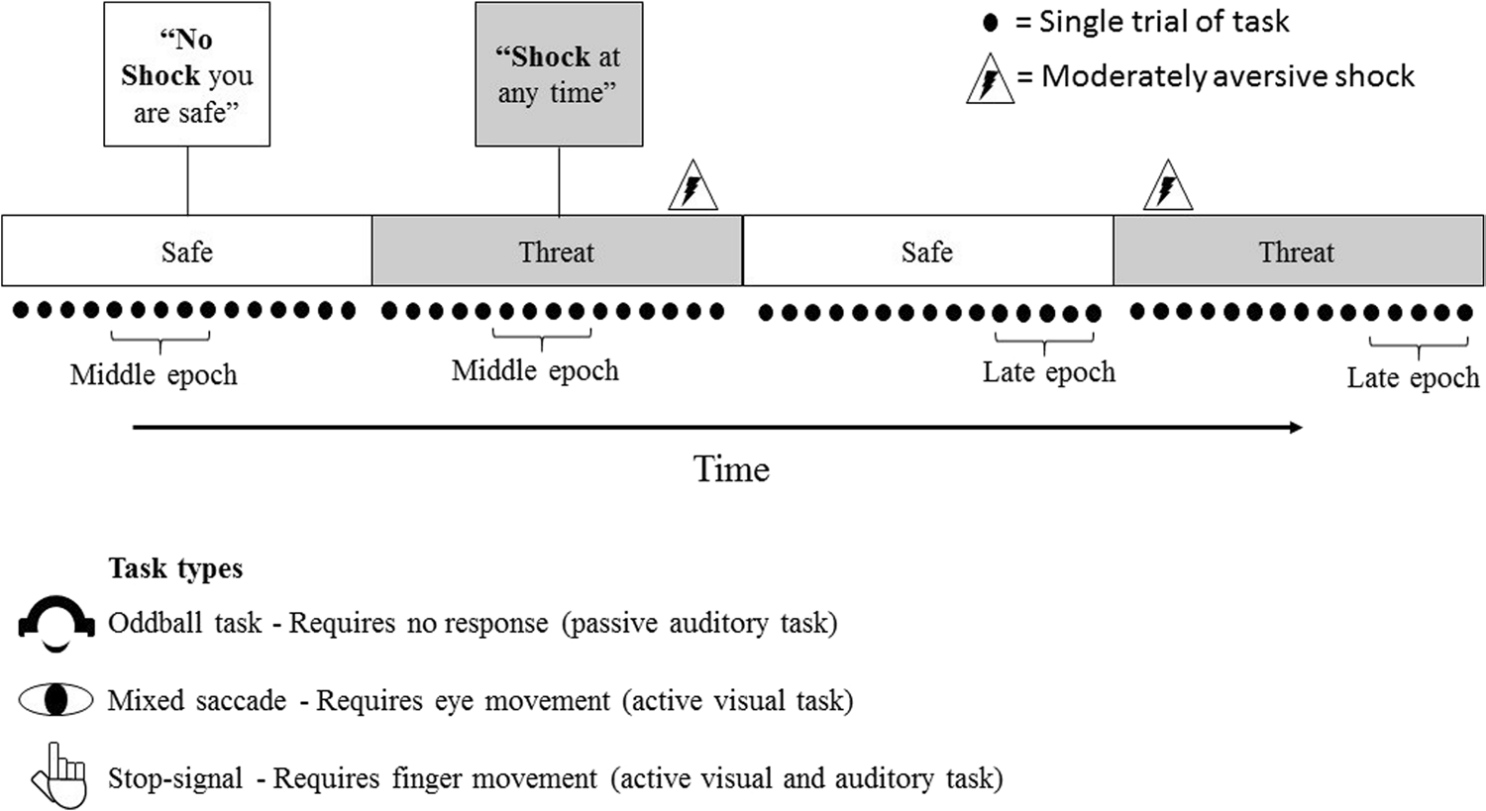
Threat of shock procedure contained alternating threat and safe conditions in all three studies. Each threat and safe block contained many trials (between 10 and 33 trials per block depending on the study). Epochs were taken from threat and safe blocks, ensuring that shocks were not captured in the epoch and epochs were not time locked to trials, stimuli, or responses to ensure nonspecific sustained anxiety was being examined

During THREAT and SAFE periods, participants engaged in one of three tasks (Fig. 1): a passive oddball task (Cornwell et al., 2007), a stop-signal task (Roxburgh et al., 2020), or a mixed-saccade task (Cornwell et al., 2012). For the mixed-saccade and stop-signal tasks, each run had five THREAT and five SAFE periods, with each block lasting approximately 70 sec. For the oddball study, each run had ten THREAT and ten SAFE periods, each lasting approximately 35 sec. In all studies, participants were instructed that the stimuli and task were unrelated to shock administration. Participants rated their subjective anxiety (out of 10) after each threat and safe block.

### MEG acquisition

Data for the oddball and mixed-saccade studies were obtained with a CTF 275-channel, whole-head MEG system (VSM MedTech, Ltd., Canada) in a magnetically shielded room (Vacuumschmelze, Germany) by using synthetic third gradient balancing for active noise cancellation. Data for the stop-signal study was obtained by using a 306-channel Elekta Neuromag^®^ TRIUX magnetometer MEG system (Helsinki, Finland) in a magnetically shielded room with internal active shielding disengaged. Magnetic flux density was digitized at a sampling rate of 600 Hz (oddball), 1200 Hz (mixed-saccade), or 1000 Hz (stop-signal). In all studies, radiological markers were placed on each fiducial position for later co-registration with individual anatomical MRIs. In all three studies, participants that exceeded 5 mm in total head displacement from the start of the scan were removed from analysis (14 removed in total).

### MEG analysis

#### Epoch extraction

The approach to epoch extraction across the three data sets was to find windows not contaminated by shocks nor time-locked to a trial, stimulus, or response. In this way, we can ask what state-related changes in neuronal population activity occur as a person experiences alternating periods of threat and safety. For the mixed-saccade and stop-signal studies, there was a total of 10 periods for THREAT and 10 for SAFE (2 runs x 5 alternations), each lasting approximately 70 sec. Two separate 10-sec epochs were extracted from each THREAT and SAFE period. Epoch timing followed the onset of a THREAT or SAFE period and was chosen to ensure shocks were not delivered during the window: for the middle epoch, timing was 29-39 sec for the SST and 27-37 sec for the mixed-saccade task; for the late epoch, timing was 55-65 sec for the SST and 48-58 for the mixed-saccade task. For the oddball study, there was a total of 20 periods for THREAT and 20 for SAFE (2 runs x 10 alternations), each lasting approximately 35 sec. Because of the shorter period, 5-sec epochs were extracted from these data (10-15 sec for the middle interval and 25-30 sec for the late interval). Thus, epochs were either 5 or 10 sec in duration and did not differ between THREAT and SAFE conditions.

#### Filtering, noise reduction, source reconstruction, and beamforming

Epochs were extracted and bandpass filtered by using a zero phase-shift, fourth-order Butterworth bandpass filter across standard frequency windows: 4-8 Hz for theta, 8-13 Hz for alpha, 14-30 Hz for beta, and 30-50 Hz for gamma. The entire recording was first bandpass filtered before epochs are extracted for covariance estimation. Noisy and flat channels were removed from the analysis; no other data/noise statistical reduction techniques were used before source analysis. Individual MRIs were used for source modelling. T1-weighted structural MRI acquired for the stop signal study used a 3-T Siemens Trio system with the following parameters: TR = 1.9 s, TE = 2.5 ms, matrix = 256 × 256, and sagittal slice thickness = 1 mm. Mixed saccade and oddball both used T1-weighted structural MRI acquired on a 3-T whole-body scanner (GE Signa, WI, USA) with the following parameters: sagittal slice thickness = 1 mm, TR = 7.24 ms, TE = 2.7 ms, matrix = 256 x 256.

##### Stop-signal study

For lead‐field calculation, the Nolte method (Nolte, 2003) was used to generate single‐shell head models from the spatially coregistered MRIs. MEG data were analysed with a linear‐constrained minimum‐variance (LCMV) beamformer method in Fieldtrip software (Oostenveld et al., 2011). A single data covariance matrix (without regularization) was calculated across all sensors (except those removed) from the bandpass-filtered data. Contrasts were made directly between spectral power during THREAT and spectral power during SAFE, resulting in estimates of relative power (log_10_-transformed power ratios).

##### Mixed-saccade and Oddball Studies

A multiple-spheres model was used to compute the forward solution. Data were analysed with synthetic aperture magnetometry (or SAM beamformer) using CTF software along with freely available software tools developed by the NIMH MEG Core facility (http://kurage.nimh.nih.gov). Contrasts were made directly between spectral power during THREAT and spectral power during SAFE, resulting in estimates of relative power (pseudo-F power ratio).

For data from all three tasks, a single data covariance matrix was calculated (without regularization) from epoch extracted, bandpass-filtered data. For all three studies, each individual source image consisted of a volume of power ratios with a spatial sampling of 5 mm. Positive power ratios represent greater power during THREAT than SAFE, and vice versa.

#### Group analyses

Group analyses were conducted by using AFNI (Cox, 1996) after transforming individual volumetric data into a common Talairach space and normalizing voxel (38,086) statistics. Differences in band power between conditions were then calculated for each window in each study. One-sample, Student *t* tests were performed on a voxel-wise basis with a test case of zero, reflecting the null hypothesis of equal oscillatory signal power between THREAT and SAFE periods for a given region. To establish spatial overlap in differential regional activity across studies, joint probability values were obtained from voxel-wise *t* statistics across the three studies and a single, false-discovery rate (FDR) calculation was performed for all frequency bands and middle and late time intervals. Joint probability values below .0019 corresponded to an overall FDR below 1%. Thus, we settled on using a nominal *p* < .05 per study to identify convergence in regional differences across studies (joint probability value < .000125 = .05 x .05 x .05).

## Results

### Decreased beta-band activity during sustained threat across three independent samples

**Fig. 2:**
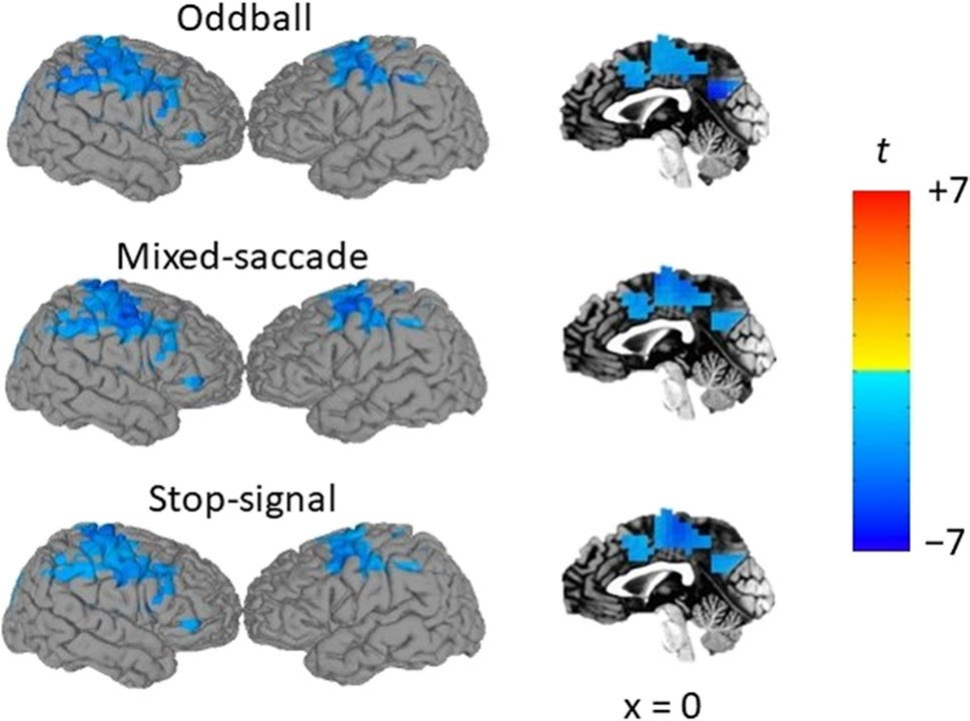
Decreased beta-band activity over sensorimotor and right prefrontal areas during sustained threat of unpredictable shock across three independent samples. Pial surface reconstructions and sagittal slices of a standardized brain are overlayed with *t* statistic maps mean beta power ratios (THREAT/SAFE, log10-transformed) over regions where mean beta power differences between conditions (THREAT – SAFE) survive a threshold of .05 for all three studies

Whole-brain adaptive beamformer analyses were performed to examine spatial convergence of differential beta-band activity (14-30 Hz) between THREAT and SAFE conditions across the three studies. Figure 2 displays common regions of differential beta during the middle interval separately for each study. As can be observed, threat-related decreases in beta power span bilateral sensorimotor cortices and right ventrolateral prefrontal cortex.

A reduction in beta power during THREAT compared to SAFE was found across all three studies in five regional clusters. The first large cluster spanned the bilateral sensorimotor areas and part of the superior and medial parietal lobe. The second cluster was in the right mid orbital gyrus. The third was localized to the right inferior frontal gyrus. Two more small clusters were in the anterior cingulate cortex. Details of clusters for middle and late windows can be found in Supplementary Material (Tables S2 and S3).

**Fig. 3:**
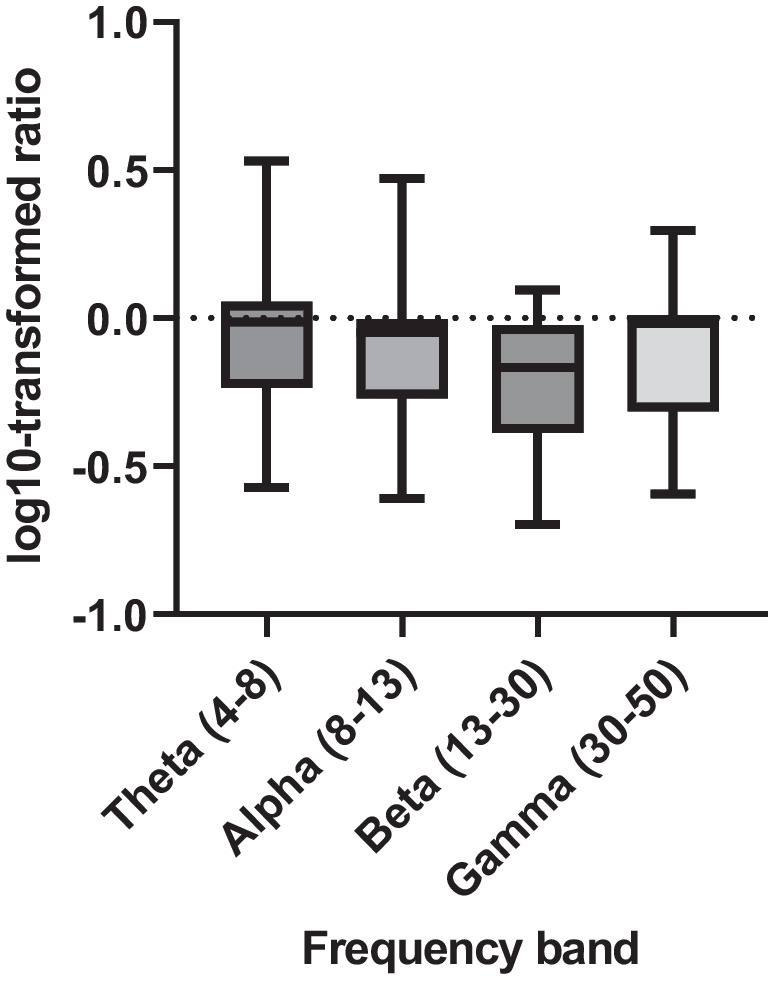
Box plots of log 10-transformed power ratios (threat-safe), showing the descriptive, post hoc spectral specificity of beta desynchronization

Individual log10 power ratios (threat-safe) for each subject for each frequency window (theta, alpha, beta, gamma) were extracted at the peak coordinates in the sensorimotor cluster to reveal spectral specificity in this region. Figure 3 shows that power desynchronization appears to be greater for beta band compared with theta, alpha, or gamma bands.

### Decreased alpha-band activity during sustained threat across three independent samples

**Fig. 4.**
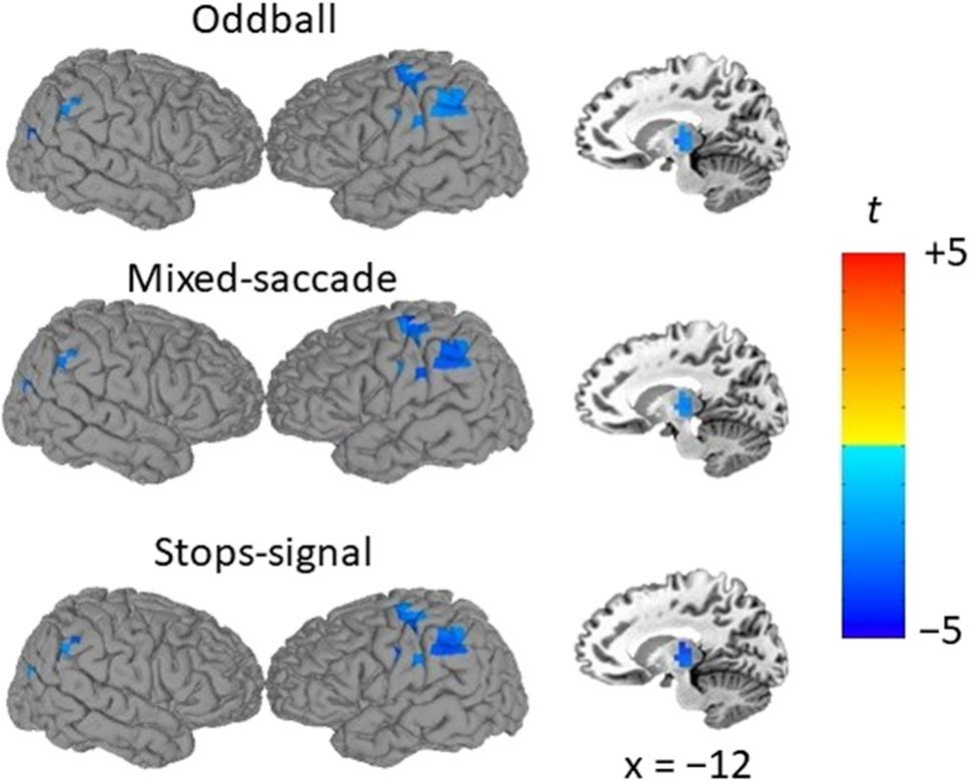
Decreased alpha-band activity during sustained threat of unpredictable shock across three independent samples. Pial surface reconstructions and sagittal slices of a standardized brain are overlayed with t statistic maps mean beta power ratios (THREAT/SAFE, log10-transformed) over regions where mean alpha power differences between conditions (THREAT – SAFE) survive a threshold of .05 for all three studies

The same procedure used to explore beta-power related changes was used to explore alpha-power changes. Figure 4 shows the common regions of differential alpha during the middle interval separately for each study. Threat-related decreases in alpha-power can be seen in the left intraparietal sulcus (IPS), thalamus, intraparietal junction, and sensorimotor areas. Cluster details for middle and late windows are shown in Supplementary Tables S4 and S5.

### No significant differences in theta-band or gamma-band activity during middle windows but decrease in sensorimotor gamma in late window

There were no significant clusters of differential theta power between THREAT and SAFE across all three studies. The same was true for gamma power in the middle interval. However, there was a threat-related reduction in gamma power in the late window over sensorimotor cortices. Cluster details are shown in Supplementary Table S6.

### Differences in subjective anxiety were not correlated with differences in oscillatory power

While subjective anxiety was significantly higher in threat compared with safe (*t*(50) = 13.4, *p* < .001; confirming anxiety was successfully induced), these differences (threat – safe) were not significantly correlated with changes in beta or alpha power (threat – safe) at the peak coordinate of the sensorimotor cluster.

## Discussion

Across three independent studies, anxiety induction was associated with a reduction in beta band power, most prominently in bilateral sensorimotor cortices. Importantly, this reduction occurred in three different task contexts, including a passive oddball task with no motor response requirements. This suggests the reduction in beta-band power is task-independent and reflects a more general anxiety state triggered by unpredictable threat. Based on the “status quo” theory (Engel & Fries, 2010) and related claims (Kilavik et al., 2013; Schmidt et al., 2019), threat-related reductions in sensorimotor cortical beta power likely reflect heightened action readiness even without overt motor actions (e.g., passive oddball listening), as a protective mechanism to cope with potential threat (Ekman et al., 1994; Robinson et al., 2013). Notably, a resting state MEG study comparing patients with PTSD to healthy controls found that patients showed decreased beta oscillatory power in a number of regions, including right superior frontal gyrus, midline supplementary motor areas (SMA), and bilateral sensorimotor cortices (Huang et al., 2014). Thus, while the current study arguably showed adaptive cortical changes in healthy individuals exposed to the threat of unpredictable shocks, similar changes may underlie anxiety pathologies as they become persistent and situationally inappropriate.

Reductions in beta power also were found over the right prefrontal areas, including the right IFG and right mid orbital gyrus. A reduction in beta power over right prefrontal regions, particularly the right IFG, may have reflected priming of stimulus-driven attention processes. Cornwell et al. (2017) found that feedforward projections to the right IFG in response to infrequent stimuli were facilitated by induced anxiety, whereas feedback projections were impaired, supporting the notion that the right IFG plays a role in the detection of deviant early sensory input (Doeller et al., 2003), which is facilitated by induced anxiety at the potential cost of later processing. A likely cost of this preference for stimulus-driven processing is impaired goal-directed processing. Increased beta power in the right IFG is generally associated with improved motor inhibition (Castiglione et al., 2019; Swann et al., 2009; Wagner et al., 2017; Wessel et al., 2013). However, we recently showed that inhibitory increases in right prefrontal beta are lessened during induced anxiety (Roxburgh et al., 2020). Our current data together with these previous findings suggest that anxiety induces a reduction in right prefrontal and sensorimotor beta power that likely facilitates action readiness at the expense of action inhibition. Indeed, increases in right prefrontal beta facilitate motor inhibition, whereas decreases in sensorimotor beta facilitate motor movements (Engel & Fries, 2010). Importantly, our study showed how anxiety-related changes could lead to cognitive deficits seen in anxiety disorders. For example, those with PTSD tend to have poorer inhibitory control (Swick et al., 2012; van Rooij et al., 2014), which may be due to heightened stimulus-driven responding.

The results also revealed a decrease in alpha power over the left intraparietal sulcus (IPS) and thalamus. The reduced alpha-power in the IPS was consistent with the findings of Balderston et al. (2017), who also showed, with MEG, a threat-induced decrease in alpha in the left intraparietal sulcus. Balderston et al. (2017) argued this reduction in alpha reflected changes in attention due to the role of the IPS in attentional control. Our findings support this assertion. However, we demonstrated that this reduction occurred regardless of task demands; it happened even during the passive oddball task where goal-directed attention was not required. Like the findings of a reduction in sensorimotor beta, this finding indicated that there were sustained anxiety-related changes in the brain that were independent of task type. This suggests that induced anxiety facilitates general changes in the preparation of possible future motor or attentional demands rather than (or in addition to) changes made in response to motor and attentional demands.

The decreased alpha found in the thalamus could reflect a node in an anxiety related network. Indeed, Balderston et al. (2017) found an increase in connectivity between the thalamus and IPS during induced anxiety compared to safe conditions. Furthermore, the thalamus has been implicated as a key node in the canonical fear network and in fear conditioning (Fullana et al., 2016). Finally, when exploring the functional connectivity of two key fear- and anxiety-related structures (bed nucleus of the stria terminalis and central amygdala) during sustained threat-induced anxiety, Torrisi et al. (2018) reported that the central amygdala became more strongly coupled to the thalamus under threat. They argued the thalamus plays a role in sensory and attentional adaptations during sustained anxiety. Furthermore, Hermans et al. (2014) argued that the thalamus is part of a network that responds to acute stress by facilitating attention. Our results support the contention that the thalamus plays a role in the brain’s response to prolonged uncertain threat and suggest that these changes exist as a threat-induced reduction in the alpha-band power. This facilitation of attention, particularly to threat, is another aspect of the hypervigilance seen in anxiety-related disorders (American Psychiatric Association, 2013; Bangel et al., 2017; Cisler & Koster, 2010).

Previous work has implicated right PFC activation in anxious sates manifested as a reduction in EEG alpha power in tasks, such as anticipating public speaking (Davidson et al., 2000), worry (Hofmann et al., 2005), and exposure to fearful faces (Avram et al., 2010). The current study did not find changes in alpha power in frontal regions during threat compared with safe. This may be for several reasons; one possibility is that task irrelevant anticipation of shock may have different underlying neural mechanisms than other aspects of anxiety. Indeed, Hofmann et al. (2005) show that worry and anticipation (of public speaking) have different frontal asymmetries. The current study used threat of shock occurring alongside task performance. In this way, arousal was likely associated with preparation of motor function, more than other aspects of anxiety, such as worry. This specific aspect of anxiety may be the reason the current study did not find frontal alpha asymmetry. Alternatively, it may be that the higher spatial resolution of MEG produced more localised changes, that frontal alpha differences may have been lost through FDR corrections, or that beamforming may have suppressed alpha differences. Future work may wish to assess specifically the changes in frontal asymmetry through more targeted analyses.

It should be noted that the pattern of differential oscillatory power varies to a moderate degree between the middle and late interval windows. While the decrease in sensorimotor cortical beta remained in both windows, the right ventrolateral prefrontal cortical beta did not. Similarly, fewer regions showed reduced activity in alpha during the late window compared with the middle window. It is possible this general reduction over time (from middle to late windows) in oscillatory power differences between threat and safe could have reflected a partial waning of anxious arousal over the threat period. Indeed, even the robust bilateral sensorimotor cortical beta difference partially waned from the middle to the late window. The general reduction in changes suggests a general reduction in the effect of the experimental manipulation. Another limitation in the data is the lack of findings in areas that show fear-related changes. For example, fear is associated with changes in theta-band power in the amygdala, hippocampus, and medial PFC (Lesting et al., 2011; Maratos et al., 2009; Pape et al., 2005). fMRI studies have implicated these structures in anxiety (Grupe & Nitschke, 2013). However, we did not find anxiety-induced changes in theta power or in these structures in any other frequency range. This may be due to the differences between anxiety and fear. The electrophysiological studies implicating these regions tended to look at short-lived responses to immediate threat (i.e., fear), while the present study focused on sustained responses to unpredictable threat. Additionally, the study did not find a correlation between sensorimotor beta desynchronization and changes in subjective anxiety between threat and safe. This may suggest that subjective anxiety is not a strong measure of anxious motor preparation (perhaps instead reflecting other aspects of anxiety such as worry). While researchers have shown that it is possible to detect deep-brain activity by using MEG, these signals are weaker and results from the current paper relating to deep-brain structures should be viewed with caution until replicated. A final limitation is that the direct contrast of two conditions only allowed for the comparison of these conditions and did not provide separate data on each condition. Thus, the nature of our analysis means that we could not determine oscillatory idiosyncrasies of each condition (e.g., is lower beta in threat due to beta desynchronization in threat, beta synchronization in safe, or a combination). Nevertheless, our results showed neural oscillatory changes that differentiate sustained anxiety from normal conditions.

The current findings of decreased sensorimotor beta may have treatment implications. For example, it is generally accepted that benzodiazepines induce a “beta buzz”—an increase in beta activity (Domino et al., 1989; van Lier et al., 2004). The current findings indicate that anxiolytic effects of benzodiazepines could be to boost beta-band signalling, counteracting the suppression of beta-band activity during anxious arousal. In addition, monitoring beta oscillatory activity in real time over sensorimotor cortices may provide a useful proxy of a patient’s current state of arousal. This could be used to inform exposure therapies, or it could be used to help patients reduce anxiety through neurofeedback techniques. Preliminary studies have attempted to increase beta activity in patients with anxiety without specifically target sensorimotor areas, reporting evidence of symptoms waning and cortisol levels dropping following neurofeedback training (Aliño Costa et al., 2016; Moradi et al., 2011)

## Conclusions

Overall, our data show that threat-induced anxiety is reflected in oscillatory changes in regions associated with action readiness and attention/vigilance. When prolonged or extreme, these state-related changes may underlie the pathological hypervigilance seen in many psychiatric disorders. These findings may aid in the detection of anxious states and in the treatment of pathological anxiety and hypervigilance. However, these findings should be extended to other anxiety provoking paradigms, such as speech anticipation. The authors urge future work to use additional anxiety-inducing paradigms to provide a more complete understanding of sustained anxious arousal.

### Supplementary Information

Below is the link to the electronic supplementary material.

Supplementary file1 (DOCX 23 KB)

## Data Availability

None of the data for the experiments reported is available, because this was not part of the ethically approved data management procedure nor was it part of informed consent. None of the experiments was preregistered.
