# Supplementary material for: A neural oscillatory signature of sustained anxiety
Source: Cogn Affect Behav Neurosci. 2023 Oct 25;23(6):1534–44. doi: 10.3758/s13415-023-01132-1 (PMC10684633; doi:10.3758/s13415-023-01132-1)
Supplement: Supplementary file 1 — Supplementary file1 (DOCX 23 KB) [file 13415_2023_1132_MOESM1_ESM.docx]

**Supplementary Material**

| Table S1. *Participant characteristics for each task* | | | | |
| --- | --- | --- | --- | --- |
|  | Number | Mean age | Male | Female |
| Oddball | 16 | 28 | 11 | 5 |
| Mixed-saccade | 17 | 27 | 10 | 7 |
| Stop-signal | 18 | 27 | 14 | 4 |
| **Total** | **51** | **27** | **35** | **16** |

| Table S2. *Brain regions showing* *differential beta-band activity between THREAT and SAFE conditions during the middle window* | | | | | | | | | | | | | | | | | |
| --- | --- | --- | --- | --- | --- | --- | --- | --- | --- | --- | --- | --- | --- | --- | --- | --- | --- |
|  |  |  | Oddball | | | | | Mixed-saccade | | | | | Stop-signal | | | | |
|  | BA | Size | Peak(xyz) | | | t statistic | *p* | Peak(xyz) | | | t statistic | *p* | Peak(xyz) | | | t statistic | *p* |
| Sensorimotor/Precuneus | 3/6/31 | 1873 | 3 | -59 | 25 | -6.80 | <.001 | 19 | -20 | 87 | -5.77 | <.001 | 19 | -20 | 57 | -5.71 | <.001 |
| Mid Orbital Gyrus | 14 | 13 | 14 | 36 | -5 | -2.87 | .006 | 14 | 46 | -11 | -2.74 | .008 | 19 | 41 | -5 | -2.94 | .004 |
| Right IFG | 45 | 8 | 51 | 41 | -5 | -2.91 | .006 | 51 | 41 | -5 | -3.24 | .002 | 46 | 36 | -5 | -2.90 | .006 |
| Anterior Cingulate | 24 | 4 | 8 | 32 | 13 | -2.29 | .026 | 8 | 27 | 13 | -2.17 | .035 | 8 | 32 | 13 | -2.54 | .014 |
| Anterior Cingulate | 33 | 4 | 8 | 23 | 19 | -2.46 | .018 | 14 | 23 | 19 | -2.50 | .016 | 14 | 28 | 19 | -2.47 | .016 |
| Note: BA = Brodmann Area, Size is in voxel counts, Coordinates are in MNI space, Sensorimotor/Precuneus is a large cluster spanning these regions, clusters required a minimum voxel number of 4 to be included in the table, joint probabilities for all regions are below .0001 and survive FDR corrections at .05 | | | | | | | | | | | | | | | | | |

| Table S3. *Brain regions showing* *differential beta-band activity between THREAT and SAFE conditions during the late window* | | | | | | | | | | | | | | | | | |
| --- | --- | --- | --- | --- | --- | --- | --- | --- | --- | --- | --- | --- | --- | --- | --- | --- | --- |
|  |  |  | Oddball | | | | | Mixed-saccade | | | | | Stop-signal | | | | |
|  | BA | Size | Peak(xyz) | | | t statistic | *p* | Peak(xyz) | | | t statistic | *p* | Peak(xyz) | | | t statistic | *p* |
| Sensorimotor | 3/6 | 1118 | 51 | 14 | 37 | -6.19 | <.001 | 35 | -7 | 67 | -7.20 | <.001 | 24 | -6 | 73 | -4.77 | <.001 |
| Note: BA = Brodmann Area, Size is in voxel counts, Coordinates are in MNI space, Sensorimotor, clusters required a minimum voxel number of 4 to be included in the table, joint probability is below .0001 and survives FDR corrections at .05 | | | | | | | | | | | | | | | | | |

| Table S4. *Brain regions showing* *differential alpha-band activity between THREAT and SAFE conditions during the middle window* | | | | | | | | | | | | | | | | | |
| --- | --- | --- | --- | --- | --- | --- | --- | --- | --- | --- | --- | --- | --- | --- | --- | --- | --- |
|  |  |  | Oddball | | | | | Mixed-saccade | | | | | Stop-signal | | | | |
|  | BA | Size | Peak(xyz) | | | t statistic | *p* | Peak(xyz) | | | t statistic | *p* | Peak(xyz) | | | t statistic | *p* |
| Middle Cingulate | 23 | 42 | 19 | -9 | 38 | -2.99 | .004 | 19 | -9 | 27 | -4.39 | <.001 | 14 | -21 | 39 | -3.73 | <.001 |
| Precentral Gyrus | 6 | 35 | -45 | -7 | 61 | -3.47 | .001 | -40 | -13 | 62 | -3.67 | <.001 | -29 | -15 | 33 | -3.61 | <.001 |
| Inferior Parietal Lobule | 40 | 28 | -67 | -31 | 40 | -3.33 | .002 | -61 | -31 | 46 | -3.59 | <.001 | -61 | -31 | 40 | -3.68 | <.001 |
| Thalamus | - | 14 | -13 | -11 | -2 | -3.13 | .003 | -13 | -24 | -1 | -2.77 | .008 | -13 | -17 | 10 | -4.07 | <.001 |
| Precentral Gyrus | 6 | 9 | -50 | -3 | 27 | -2.91 | .005 | -50 | -9 | 27 | -2.66 | .010 | -56 | -3 | 32 | -3.59 | <.001 |
| Postcentral Gyrus | 2 | 9 | 40 | -31 | 76 | -2.17 | .035 | 35 | -31 | 52 | -3.20 | .002 | 30 | -26 | 45 | -2.87 | .006 |
| Note: BA = Brodmann Area, Size is in voxel counts, Coordinates are in MNI space, Sensorimotor/Precuneus is a large cluster spanning these regions, clusters required a minimum voxel number of 4 to be included in the table, joint probabilities for all regions are below .0001 and survive FDR corrections at .05 | | | | | | | | | | | | | | | | | |

| Table S5. *Brain regions showing* *differential alpha-band activity between THREAT and SAFE conditions during the late window* | | | | | | | | | | | | | | | | | |
| --- | --- | --- | --- | --- | --- | --- | --- | --- | --- | --- | --- | --- | --- | --- | --- | --- | --- |
|  |  |  | Oddball | | | | | Mixed-saccade | | | | | Stop-signal | | | | |
|  | BA | Size | Peak(xyz) | | | t statistic | *p* | Peak(xyz) | | | t statistic | *p* | Peak(xyz) | | | t statistic | *p* |
| Middle Temporal Gyrus/temporal-parietal junction | 21/39 | 48 | 46 | -50 | 7 | -5.10 | <.001 | 40 | -44 | 18 | -3.63 | <.001 | 51 | -44 | 12 | -3.37 | .001 |
| Middle cingulate cortex | 24 | 17 | 3 | -8 | 44 | -2.76 | .008 | 3 | -27 | 57 | -2.62 | .012 | 8 | -15 | 39 | -3.45 | .001 |
| Pre/postcentral gyrus | 40 | 6 | -29 | -20 | 57 | -2.57 | .014 | -24 | -30 | 57 | -2.47 | .016 | -40 | -20 | 51 | -4.13 | <.001 |
| Pre/postcentral Gyrus | 4 | 13 | -40 | -9 | 38 | -3.00 | .004 | -50 | -8 | 44 | -2.79 | .008 | -50 | -20 | 45 | -2.85 | .006 |
| Postcentral Gyrus | 2 | 6 | 61 | -9 | 27 | -2.46 | .016 | 61 | -16 | 27 | -2.37 | .022 | 61 | -9 | 33 | -3.89 | <.001 |
| Note: BA = Brodmann Area, Size is in voxel counts, Coordinates are in MNI space, Sensorimotor/Precuneus is a large cluster spanning these regions, clusters required a minimum voxel number of 4 to be included in the table, joint probabilities for all regions are below .0001 and survive FDR corrections at .05 | | | | | | | | | | | | | | | | | |

| Table S6. *Brain regions showing* *differential gamma-band activity between THREAT and SAFE conditions during the late window* | | | | | | | | | | | | | | | | | |
| --- | --- | --- | --- | --- | --- | --- | --- | --- | --- | --- | --- | --- | --- | --- | --- | --- | --- |
|  |  |  | Oddball | | | | | Mixed-saccade | | | | | Stop-signal | | | | |
|  | BA | Size | Peak(xyz) | | | t statistic | *p* | Peak(xyz) | | | t statistic | *p* | Peak(xyz) | | | t statistic | *p* |
| Superior frontal/precentral gyrus | 6 | 224 | 19 | -12 | 79 | -5.95 | <.001 | 14 | -13 | 62 | -5.51 | <.001 | 19 | -6 | 79 | -3.64 | <.001 |
| Superior frontal gyrus/precentral gyrus | 6 | 8 | -29 | -6 | 73 | -2.40 | .020 | -34 | -19 | 68 | -3.48 | .001 | -29 | -7 | 67 | -3.35 | .001 |
| Note: BA = Brodmann Area, Size is in voxel counts, Coordinates are in MNI space, Sensorimotor/Precuneus is a large cluster spanning these regions, clusters required a minimum voxel number of 4 to be included in the table, joint probabilities for all regions are below .0001 and survive FDR corrections at .05 | | | | | | | | | | | | | | | | | |
